# Book Notices and Reviews

**Published:** 1864-08

**Authors:** 


### BOOK NOTICES AND REVIEWS.

*The Principles and Practice of Obstetrics:* Illustrated with One Hundred and Fifty-Nine Lithographic Figures from Original Photographs and with numerous Wood Cuts. Hugh L. Hodge, M. D., Emeritus Professor, etc. Philadelphia: Blanchard & Lea. 1864.

When we consider the number of valuable works upon Obstetric science and art which have been issued from the medi cal press within the last very few years, a doubt may arise in the mind, whether a new work upon this subject was so soon demanded. But when we are apprised of the fact that the eminent author of the work before us was about to retire from the prominent position which he had so long filled with marked ability, nothing could be more proper than that he should leave, as a legacy to the profession, the rusult of his extensive experience in the department of which he has stood at the head for at least a quarter of a century.

The rare endowments of Prof. Hodge for the task, his long and successful professional career, both as a practitioner and a teacher, led the profession to expect, as the result of his life labor, a work of more than ordinary value, and we have no hesitation in saying, that all who rise from a careful perusal of its pages, will concur in the opinion, that this expectation has been realized.

The work is published in quarto form. A leading feature will strike the reader in the lithographic figures from original photographs which illustrate more perfectly than the ordinary wood-cuts ever did, the anatomy of the parts involved, and the mechanism of labor. The mechanical execution throughout is fully equal to the best work, for which the enterprising publishers are so justly celebrated.

The preface contains a succinct history of Obstetric science in this country, by which we are strongly impressed with the rapid progress which it has made during the last few years. A comparison of the work now under consideration, with the once-valued treatise of Prof. Dewes, will illustrate very well the contrast between the past and the present.

The anatomy of the pelvis, and of the fœtal skeleton, are elaborately and admirably given, and the organs of generation are well described. Many readers will think that more attention given to the description of the formation and early development of the ovum would have rendered the work more complete. The origin of the chorion and amnion is dismissed by a brief paragraph. And the student will be left in some uncertainty whether the author holds the views of Hunter in regard to the formation of the decidua, or those of "most modern physiologists."

The author joins issue with those who believe in "superfœtation," for says the text, "We have no hesitation in declaring that superfœtation in its strict definition never occurs."

Had we space we would gladly give an analysis of the work. In the arrangement there is nothing strikingly novel. We concur most fully in the author's estimate of the paramount importance of a thorough knowledge of the "mechanism of parturition," and consider the large space devoted to its illumination well employed.

The rules for Obstetric operations are clear and explicit. Upon embryulcia we are pleased to read, "In the improved state of the science of Obstetrics at the present time, the necessity of this terrible operation very seldom occurs."

A few extracts taken at random will show the author's style and treatment of certain subjects. Dr. Hodge recommends the Obstetric forceps, not merely as levers and tractors, but also as compressors:

"If," he says, "the head be large or the straits contracted, more pressure becomes requisite; otherwise delivery would be impracticable. How far this compression may be carried with safety to the infant, is a question of great interest; but the solution of it cannot have much influence on our practice, inasmuch as we must be regulated not so much by the idea how much compression can be borne with impunity, but how much is absolutely demanded to accomplish the delivery, for, of course, no more pressure should be made than what is essential for delivery. It is to be remembered, also, that the injury to the child depends much upon the longer or shorter continuance of this compression, and doubtless, also, on the degree of ossification which may exist in the cranial bones. Moreover, every practitioner is familiar with the wonderful fact, that while the placental functions continue, the brain will bear for a long time great pressure with impunity,

"We know that owing to the moulding of the head by the passages of the pelvis, and the powerful contractions of the uterus, the head may be greatly diminished laterally, and increased in length by the yielding and overlapping of the bones, in many instances, without destroying life, and we know, also, that in many cases where such compression has existed, forceps have effected a delivery, and the child been preserved. In one case of contracted pelvis, where the sacro-pubic diameter measured but little if any more than three inches, the author delivered an infant alive, whose head, a few hours after delivery, measured three inches and ten lines, in transverse diameter. We think, therefore, that the limit to be prescribed to the use of forceps as compressors must be restrained by the necessities of the case, rather than the effect it may have upon the life of the infant; for though that practitioner must be regarded as careless and even criminal who makes more compression than is absolutely necessary, yet he is fully justified in making that degree of compression requisite to effect delivery provided there be any reasonable expectation of preserving the life of the child.

"Although, therefore, the child must, in many instances, be exposed to some danger from the use of the forceps as compressors, yet they afford as fair prospect for its safety in bad cases, where otherwise it must have perished, and in most instances they are all important to diminish the sufferings and danger of the parent, with little or no risk to the infant."

The treatment of placenta prævia is perfect rest in bed, hips elevated, head low; a simple and easily digested diet; cold applications to the uterine regions, and warm to the extremities; avoidance of all mental and moral excitement. The following summary gives a good idea of the author's mode of managing placenta prævia:

"The nature of the hemorrhage being ascertained, a soft sponge, dipped in cold water and vinegar, should be applied directly over the orifice of the uterus, and supported by other portions introduced between it and the floor of the pelvis, or, perhaps, by a gum-elastic bag distended with water. If the hemorrhage should continue and the os remain rigid, the employment of the sponge tent or the internal caoutchouc dilator may possibly be advantageous before more decided ulterior treatment is employed. If, however, the hemorrhage be, by these means, diminished, and the patient's strength is good, the practitioner should wait until the os uteri be dilatable. Then, if the membranes do not rupture, they should be perforated, and the liquor amnii evacuated. If the contractions of the uterus be not powerful, the ergot should be given in repeated doses, while cold applications should be made to the uterus and rectum. If now the bearing-down efforts be efficient, and the perineum becomes distended, the tampon may be gradually removed, and, if necessary, the forceps applied to complete delivery. If the head be too large, or the pelvis small, perforation of the cranium may, in some rare instances, be demanded.

"If, unfortunately, these measures do not sufficiently diminish the hemorrhage, and the patient's strength fails, and the labor cannot be rapidly completed, a still further hope remains by at once extracting the whole placenta, either by means of the hand, or some suitable instrument, so that, if possible, the hemorrhage may be arrested and time gained for the patient's system to react, that delivery may be subsequently effected.

"If it should be ascertained that the presentation is preternatural, the membranes ought not to be ruptured until the os uteri is dilatable, so that version may be accomplished with less difficulty. In such cases, also, unless the necessity be imperative, the artificial abstraction of the placenta should be avoided, as the subsequent operation of version would be more painful and dangerous."

We have examined Prof. Hodges work with great satisfaction; every topic is elaborated most fully. The views of the author are comprehensive, and concisely stated. The rules of practice are judicious and will enable the practitioner to meet every emergency of Obstetric complication with confidence. Having said this much, it is unnecessary to advise the profession in explicit terms to obtain this work.

For sale by S. C. Griggs & Co, Price $13.

*The Pathology and Treatment of Venereal Diseases, including the Results of Recent Investigations upon the Subject.* By Freaman J. Bumstead, M. D. New and Revised Edition. Blanchard & Lea : Philadelphia.

We are glad to record the appearance of a new and improved edition of the very excellent work of Dr. Bumstead, now three years in the hands of the profession. There is, perhaps, no class of diseases whose proper treatment is a matter of greater moment to the patient than the venereal, and of few has medical literature and medical teaching left us in greater doubt. No writer has done so much as Dr. Bumstead, to clear up this obscurity and relieve the subject from its former confusion. He has made a plain distinction between chancre and chancroid, and teaches that from chancre constitutional syphilis is liable to supervene, but that chancroid ulcers, though contagious, are never followed by such symptoms. As a result of the recognition of this truth follow recommendations of treatment that are highly conservative, and that the experience of the profession has gone far to approve.

His use of mercurials is limited to cases of true syphilis, and never extends to the treatment of chancroid. On this point we quote, "The internal use of mercury has no beneficial influence whatever upon the chancroid, which continues in a state of stubborn persistency, or even progresses, after the system is fully under the influence of this mineral. This statement is not a mere inference from the distinct nature of the chancroid and syphilis, but is founded upon experience. I was fully convinced of the fact by personal observation, and ceased to employ mercury for "soft chancres," several years before the distinction between the two species was recognized. Since abandoning it in my own practice, I have had numerous opportunities of observing other physicians administer mercurials for the chancroid, and my former opinion has only been confirmed."

An indistinct idea once prevailed that mercury was a specific for venereal disease, and this led many to its indiscriminate use, and not only chancre and chancroid, but gonorrhœa was sometimes treated by ptyalism. Dr. Bumstead's work is so generally and favorably known as to require no praise from us, yet we do not hesitate to add, that on the subjects of which it treats it is by far the best book in the language.

For sale by W. B. Keen & Co. Price $4.50.

---

*The Physician's Dose and Symptom Book*, Containing the Doses and Uses of all the Principal Articles of the Materia Medica and Officinal Preparations. By Joseph H. Wythes, A. M., M. D., Author of "The Microscope," "Curiosities of the Microscope," etc. Fourth Edition. Philadelphia: Lindsay & Blakiston. 1864.

The title-page indicates the character and scope of the book. Having rapidly passed to the fourth edition proves the favor which it has received from the profession. Compiled for the assistance of students it is a convenient *vade mecum* for the general practitioner. The following are the contents:

Table of Weights and Measures. Rules to Proportion the Doses of Medicine. Common Abbreviations used in Writing Prescriptions. Table of Poisons and Antidotes. Classification of the Materia Medica. Dietetic Preparations. Table of Symptomatology. Outlines of General Pathology and Therapeutics.

---

*Scanzoni's Practical Treatise on the Diseases of the Sexual Organs of Women.*

We have recently been favored, by the Publishers, with a copy of this valuable work. It affords us great pleasure to state that this book is accessible to the American practitioner. It will be found by a perusal that, while the teachings are eminently conservative, the treatise embraces the latest doctrines of the pathology, and the most approved mode of treating the important diseases peculiar to woman. The work is comprehensive; its style concise, rendering it most valuable to the student and practitioner. In every particular it is worthy the celebrated Würzburg Professor.

The American Editor, Dr. A. K. Gardner, has performed his labor well, and in mechanical execution the book is a model.

